# The relation of culture, socio-economics, and friendship to music preferences: A large-scale, cross-country study

**DOI:** 10.1371/journal.pone.0208186

**Published:** 2018-12-14

**Authors:** Meijun Liu, Xiao Hu, Markus Schedl

**Affiliations:** 1 Division of Information and Technology Studies, University of Hong Kong, Hong Kong, China; 2 University of Hong Kong Shenzhen Institute of Research and Innovation, Shenzhen, China; 3 Institute of Computational Perception, Johannes Kepler University Linz, Linz, Austria; University of Zurich, SWITZERLAND

## Abstract

Music listening is an inherently cultural behavior, which may be shaped by users’ backgrounds and contextual characteristics. Due to geographical, socio-economic, linguistic, and cultural factors as well as friendship networks, users in different countries may have different music preferences. Investigating cultural-socio-economic factors that might be associated with between-country differences in music preferences can facilitate music information retrieval, contribute to the prediction of users’ music preferences, and improve music recommendation in cross-country contexts. However, previous literature provides limited empirical evidence of the relationships between possible cross-country differences on a wide range of socio-economic aspects and those in music preferences. To bridge this research gap, and drawing on a large-scale dataset, LFM-1b, this study examines the possible relationship between cross-country differences in artist, album, and genre listening frequencies as well as the cross-country distance in geographical, socio-economic, linguistic, cultural, and friendship connections using the Quadratic Assignment Procedure. Results indicate: (1) there is no significant relationship between geographical and economic distance on album, artist, and genre preferences’ distance at the country-level; (2) the cross-country distance of three cultural dimensions (masculinity, long-term orientation, and indulgence) is positively associated with both the album and artist preferences distances; (3) the between-country distance in main languages has a positive relationship with the album, artist, and genre preferences distances across countries; (4) the density of friendship connections among countries negatively correlates to the cross-country preference distances in terms of artist and genre. Findings from this study not only expand knowledge of factors related to music preferences at the country level, but also can be integrated into real-world music recommendation systems that consider country-level music preferences.

## Introduction

With the popularity of music available online through worldwide streaming services, growing importance has been attached to exploring cross-country similarities and differences in music taste within the field of music recommendation and retrieval [1–3]. Gaining the knowledge of country-specific preferences allows the personalization of music retrieval and recommendation systems on an individual, as well as on a group level. In fact, recent advances in music retrieval and music recommendation systems research have demonstrated that users’ background and contextual information is essential for personalized results [4–7]. Users’ contextual information is defined as various external factors that impact how a listener perceives music [8], which can be divided into five groups, i.e., environment context, personal context, social context, and spatial-temporal context [9]. Users' context information is important to enable personalized music services that are crucial to the future of Music Information Retrieval (hereafter MIR) [8, 10]. Geographical, economic, linguistic, cultural, and social network factors are perceived as critical aspects of user context [2, 5, 11, 12]. In this paper, we conduct a study that analyses not only the differences in music tastes between countries on a large scale, but also sets out to uncover the relationships between the country differences in the aforementioned factors and those in music preferences.

A long-standing view in the literature is that an individual’s patterns of tastes, opinions, and consumption of cultural objects can reflect a broader vision of moral, social, and cultural values [13]. Music listening is an important component of cultural behavior. There are, however, ongoing discussions about the cross-culture or cross-country disparity in music information behaviors, such as music search, management, consumption, as well as music mood perception [1, 14–16]. The literature on music preferences has also studied cross-country differences from cultural and socio-economic perspectives [4, 17]. Addressing these questions can promote the construction of cross-country/cultural music retrieval and recommendation systems, and improve recommendation and retrieval results by taking the cultural and socio-economic background of users into account [4]. From a global and multicultural perspective, this line of research also contributes to enriching the knowledge of cross-country difference as evidenced in customs, traditions, cultural values, and other socio-economic indicators.

To date, the literature provides limited empirical evidence of the relationship between cross-country differences in music preferences and a wide range of socio-economic aspects, e.g., language, culture, and income. Early studies investigated the relationship between socio-cultural-economic factors, e.g., social connections [18], cultural traditions [19], social class [20], educational level [20] and languages [21], and individuals’ music preferences. However, these studies suffered limitations due to small sample sizes and self-reported data. More importantly, these studies usually focused on a single cultural or national context, whereas whether the relationship exists in the context of multiple countries remains unknown. Although in some recent cross-country studies, the relationship between one or two aspects of socio-cultural-economic factors (e.g., the economic aspect [22], the cultural aspects [23], and friendship connections [24]) and music listening has been explored, many important factors have not been included in the models, which may lead to unreliable results [25, 26]. This is because missing relevant variables in statistical models may bring omitted variable bias. In other words, the estimator becomes inaccurate if the omitted variable is correlated with at least one of the explanatory variables. Besides which, previous studies have not attempted to explore the differences between album, artist, and genre preferences. In other words, it is unclear whether the between-country differences in socio-cultural-economic factors that are related to the between-country differences in album or artist listening can also explain the between-country differences in genre listening or not.

Inspired by these research gaps, this study aims to examine whether cross-country differences in *music taste represented by artist*, *album*, *and genre preferences* are related to a range of cultural and socio-economic factors. We do so through a series of quantitative analyses including descriptive analysis and the Quadratic Assignment Procedure (QAP), on a *large-scale dataset of listening events from around the world*, i.e., LFM-1b dataset [27]. The dataset contains listening histories of a large quantity of users obtained from the online music service Last.fm. Compared to self-reported data, or data collected in user experiments, this dataset could provide a naturalistic and relatively objective picture of users’ listening behaviors and preferences.

The remainder of this paper is organized as follows. First, we discuss existing literature on music preferences, as well as cultural and socio-economic factors (Section 2), from which a theoretical framework is constructed for formulating our research questions and hypotheses (Section 3). Next, the data source used in the study and the methods applied are detailed (Section 4). Results of our analysis and a detailed discussion that answers the research questions are provided in Section 5. Robustness check based on world vector technique is presented in Section 6. The paper is rounded off by a conclusion that also indicates the limitations of the study (Section 7) and an outlook to future work.

## Related work

This study investigates the relationship between various cultural-socio-economic factors and music listening behavior, with a focus on the country-level geographic location, economic status, cultural dimensions, language, and density of cross-country friendship relationships. Corresponding related work is therefore composed of studies that address the relationship between these cultural-socio-economic factors and music taste.

### Geographic location and music preference

In recent years, despite increasing attention paid to users’ geospatial context for music recommendation [7, 28, 29], there are few studies on the association between geographical factors and users’ music preferences. Some recent research has suggested that the geographical information of listeners can promote the quality of music recommendation [30]. Although geographical proximity is often connected to cultural similarity, it is not always the case. For example, although located geographically close to each other, people in North Korea and South Korea may show distinct music preferences and it has therefore been suggested that combining cultural, and geographical distances may better explain differences in music taste [28].

### Economic status and music preference

Economics status can shape music preferences. Existing literature has documented that cultural consumption has a close relationship with individuals’ social status, which is frequently measured by income. Bourdieu’s class theory suggests that knowledge and appreciation of highbrow/elite culture (e.g., ballet, or opera) depends on the cultural capital people have, which is evidenced by the finding that family income during a person’s childhood plays a crucial role in the formation of their tastes [31]. People from more affluent economic backgrounds have more opportunities to visit cultural institutions and events such as museums, concerts, and theaters, and thus tend to have more knowledge and appreciation of highbrow culture [32–34]. In the field of music, the link between economic status and music preferences also exists in that music taste varies with people’s income. For example, it has been suggested that people from high-income backgrounds prefer classical music more than those from low-income backgrounds [35]. There is a difference in musical taste between people from upper-income backgrounds and people from lower-income backgrounds or those with a lower level of education [36]. Some studies have also demonstrated that individuals with high socio-economic status have a more open attitude towards new music [13]. While the influence of income on individual’s music preferences has been discussed, few studies have focused on this relationship at the country level, Woolhouse and Bansal’s study is an exception, which provides evidence of a significant correlation between countries’ Human Development Index values (this value is calculated based on life expectancy, adult literacy and school enrollment, and Gross National Income per capita), and music-download variability [22]. Some researchers have found that people’s cultural capital varies across countries [37, 38]. For example, a well-known comparative study [39] found that upper-middle-class Americans tended to narrow the distance to people in lower status by accepting popular culture while sharper boundaries existed between people in different statuses in France. Therefore, economic distance between countries may help explain some between-country differences in music preferences.

### Culture and music preference

Culture is a well-discussed factor in music information research, compared to other socio-economic aspects. It is well-acknowledged that individual taste is not fortuitous but rather is influenced by cultural standards [40]. It is argued that the formation of a person’s general behaviors and preferences largely relies on culture [41]. In the domain of music retrieval and recommendation, taking cultural factors into consideration has been a useful strategy to investigate users’ music needs at the country level [11, 23] and retrieval methods that take such cultural differences into account are considered highly desirable [14]. Some researchers integrated personal listening habits with countries’ socio-cultural-economic factors to measure listeners’ similarity and found a set of clusters where each cluster consists of a group of countries which share common music listening patterns and common cultural characteristic [42]. A recent study by Ferwerda et al. explained the between-country differences in music listening by means of six cultural dimensions proposed in Hofstede’s cultural dimensions theory [23, 43]. However, whereas Ferwerda et al. only considered the cultural factors, this current study investigates these factors in conjunction with other socio-economic features at the same time, aiming to reveal the relative importance of these factors in forming cross-country differences in music tastes.

### Language and music preference

Research involving linguistic elements in the domain of music has been traditionally concerned with lyrics, but language factors can also result in differences in music appreciation and preferences. Using small-size survey data, Abril found a significant positive correlation between familiarity with a language and attitude toward the language in song lyrics [44]. Moreover, the empirical analysis also indicated that children might respond to foreign-language songs negatively [45]. Evidence from other studies has shown that English-speaking students liked pop songs performed in English better than those with Spanish or Chinese lyrics [21]. Therefore, the dominant language of a country may relate to music exposure and possibly the collective music preference of listeners in this country as previous studies implied, a factor that will also be examined in this study.

### Friendship connections and music preference

Prior studies have demonstrated that friendship connections among listeners influence music preferences, especially for adolescents and young adults [46–48]. According to categorization in social identity theory [49, 50], individuals usually classify themselves by the music they listen to, and are also classified by others based on their music tastes [51]. For example, North and Hargreaves observed that music is utilized as a “badge” of one’s inter- and intragroup self-definition [52]. In addition, adolescents and young adults tend to like music that their friends listen to [47, 48]. These findings suggest the social functions of music and thus social relationships may be a factor of music preferences. However, these studies are traditionally built on questionnaire data, only covering specific user groups with small sample sizes. Although the Experience Sampling Method (ESM) [53] can provide better granularity and accuracy, the sample sizes in ESM is still incomparable to datasets based on listening logs, such as the LFM-1b.

There are several research gaps in the existing literature. Three strands of relevant literature discussed socio-cultural-economic factors and music preferences. The first strand directly investigates the association between socio-cultural-economic factors, e.g., income, social connections (such as parents, teachers [18] and peers [54]), cultural traditions [19], social class [20], educational level [20], and languages [21]), and music preferences at the individual level and usually focuses on a single cultural or country context [13]. However, these studies often suffer the drawbacks of small sample sizes and self-reported responses. More importantly, whether the relationship between socio-cultural-economic factors and music preferences still holds in the context of multiple countries or cultures remains unknown, considering individuals with different countries or cultural backgrounds may have different characteristics.

The second strand directly explores the relationship between one or two aspects of socio-cultural-economic factors and music listening in the context of multiple countries, while missing important factors may lead to unreliable results [25, 26]. For example, some studies only focus on the economic aspect (or human wellbeing) [22], the cultural aspects [23], or friendship connections [24].

In the third kind of literature, researchers proposed new music recommendation approaches that combine users’ contextual information, e.g., geographic locations [28, 30], cultural aspects [42, 55], and found that these techniques outperform traditional methods. However, the relationship between socio-cultural-economic aspects and music preferences was not directly explored, neither was the extent to which these factors were related to music preferences.

Besides, the existing literature largely ignores the different levels of music listening, i.e., album, artist and genre listening when exploring the relationship between socio-cultural-economic aspects and music preferences. Album, artist and genre represent music on different levels and are important entities in music retrieval and recommendations. However, the similarity or difference of music listening behaviors represented at the three levels remains to be investigated. Given the limitations in the existing literature, which socio-cultural-economics aspects, and to what extent these aspects account for the between-country differences in music preferences, are unclear, neither are the differences between album, artist and genre listening when exploring this relationship.

To fill the research gap and complement existing studies with a large-scale analysis carried out with real-world data, we set out to comprehensively explore music preferences at a country level as well as music preference relationships with geographic, socio-economic, cultural, linguistic, and friendship factors. Our preliminary study [56] already discovered the relationship between between-country differences in music *artist* preferences and the differences in several socio-economic aspects among countries.

There are some novelties in the current study compared to our previous efforts. This paper proposes a theoretical framework based on several prominent theories in sociology and psychology (Bourdieu’s class theory, Social identity theory and Hofstede’s cultural dimension theory), which was not in the previous paper [56]. This current study considerably extends our previous one [56] by including *album* and *genre* preferences of listeners in different countries to make comparisons at different granularity levels. Many studies in the previous literature [3, 57, 58] investigated one or two aspects of music listening behaviors, while falling short in capturing a comprehensive picture of users’ music preferences. Furthermore, these studies did not investigate the differences between album, artist, and genre listening. As all three constitute important entities in music collections and recommendation systems, we assume that album, artist, and genre listening indications each carry information at different but complementary levels. Intuitively, album and artist directly reflect some country or cultural information, i.e., the publisher of albums, the main language, ethnicity, and the nationality of the performers. However, genre does not directly include this kind of information. As some researchers stated, there is a tendency for people to be increasingly open to appreciating all genres [13, 59]. Nevertheless, recent research showed that even very little data about a listener’s artist and genre preferences is sufficient to predict his or her country of residence with high accuracy [60]. Therefore, it seems reasonable to assume that also genre can provide hints of the listener’s cultural background.

In this study, we provide multiple evidence to show that the between-country difference in album and artist listening may be relatively larger than that in genre listening. The results of QAP show that the between-country differences in album and artist listening may be explained by the between-country differences in some cultural dimensions, whereas the between-country differences in genre listening may not be. Considering the fact that the sparsity of the album and artist listening data may lead to unreliable measures of the between-country differences in music listening and thus hamper the reliability of the regression results, we used the word2vec technique (which is known to be effective in alleviating data sparsity) to confirm the robustness of the main findings. Our findings provide new insights in understanding music preferences that may facilitate music recommenders through considering the differences between album, artist, and genre listening.

Moreover, the study at hand analyzes the between-country differences in various socio-cultural-economic aspects and album, artist, and genre listening, which deepens our knowledge of the cross-country differences. The matrices of the cross-country differences in a wide range of socio-cultural-economic aspects and music preferences, which we computed, can be incorporated in algorithms used in current music recommenders to add a cultural component. For instance, they could be integrated as individual user factors in users’ preference vectors and factorized in a model-based approach [55]. Alternatively, in a memory-based recommendation approach (more precisely, user-based collaborative filtering), country-specific similarities could be integrated by re-weighting the user similarities according to their socio-cultural-economic proximity.

In addition, building on social identity theory, we here propose an approach to gather data about friendship connections within and between countries, and we add the density of these friendship connections as a new variable in our investigations, which reveals the association between friendship networks and music preferences in the context of multiple countries.

## Theoretical framework and research questions

**Fig 1:**
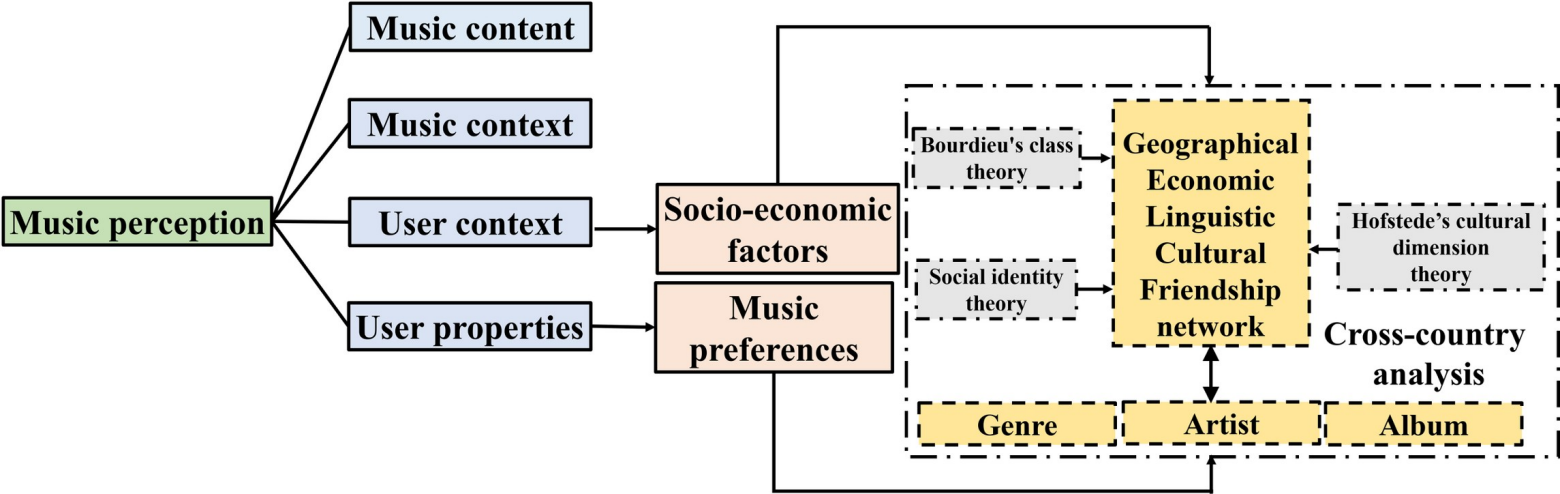
The theoretical framework of this study.

Based on established theories and models, we have constructed a theoretical framework to guide this study, which is presented in Fig 1. According to Schedl and Stober [61], and the reciprocal feedback model [62], there are four main elements that impact (and be influenced by) human music perception: music content, music context, user properties, and user context. Music content refers to information encoded in, or extracted from, the audio signal, such as rhythm patterns, melody, or instrumentation. Music context refers to information beyond, but nevertheless related to, a music item, such as the political background of the songwriter or performer, the design of an album cover artwork, or style of a music video clip. User properties describe individual user background variables, such as demographics or musical training, while user context relates to the situational dynamics of listeners, such as location, mood, or social connections. The reciprocal feedback model not only indicates important personal (e.g., demographics and musical knowledge), musical (e.g., complexity and familiarity of the music) and situational (e.g., social and cultural contexts, and everyday situations) variables which impact music perception, but also incorporates the reciprocal causal influences of all variables included in the model. In this study, music preference is a core aspect of user properties [5], and is measured by album, artist and genre listening frequency. The socio-economic factors in this study are considered important to describe user context, including geolocation [61], economic status via Bourdieu's class theory [31], language spoken in a country, cultural background [43], and friendship network [49]. These lay a solid theoretical foundation for probing the potential relationships between music preference and a variety of cultural-socio-economic factors.

Based on the above framework, we formulate the following research questions:

RQ1: What are the between-country differences in a wide range of cultural-socio-

economic aspects and in album, artist and genre listening preferences?

RQ2: To what extent can the between-country differences in album, artist and

genre preferences be predicted by the cultural-socio-economic factors?

To answer RQ2, we will use geographic location, economic development, language, culture, and friendship connections to present the between-country differences in cultural-socio-economic factors.

Geography plays a major role in shaping human behavior and international relationship [63]. Geographic locations of countries can present not only information of their physical location, but also countries’ cultural, political, and economic links [64, 65]. Additionally, some user-specific information is normally not available on online music platforms (e.g., ethnicity or religion). In such cases, geographic locations of users may provide clues to users’ cultural information, facilitating the improvement of music recommendation systems. Given these considerations, the geographic location of countries is incorporated in this study.

The consumption of cultural products or service is closely linked to economic development [66]. Residents’ consumption structure is considered optimized and upgrading with the improvement of national income level [67]. Economic development of countries encompasses both growth and welfare values, which is concerned with various socio-economic indicators (e.g., people’s entitlements, education and living standard) [68]. Hence, countries’ economic development is included as a comprehensive and representative indicator of countries’ socio-economic aspects.

Language reflects, and is shaped by, culture [69]. It is also the major means of social communication [70]. Speaking different languages is often attributed to different countries of origin, or different cultural backgrounds [70]. Given the importance of language in human life, and the inseparate relationship between language and culture, we regard the dominant language of countries as a key factor to reflect the cultural-socio-economic distance between countries.

Cultural aspects of countries encompass a wide range of concepts, e.g., belief, behavior, attitudes, values, and customs shared by people [71]. Hofstede proposed six dimensions to differentiate national cultures and established value scores on these dimensions for countries, which allows some cross-country comparisons [72]. Since Hofstede’s cultural dimension theory is generally accepted as the most comprehensive framework of national cultures, we used the value scores on six cultural dimensions for countries to measure the between-country distance in cultures.

Social relationships are essential parts of social aspects, serving important social functions [73]. As a crucial facet of social relationships, friendship ties on social network sites can reflect users’ online interaction, communication, and information sharing [74]. In the recent years, the role of social networks in music discovery and recommendation has been increasingly emphasized [75]. Therefore, friendship connections can not only be a useful indicator to measure the between-country distance in online social interaction, but also provide insights into the ongoing discussion about the relationship between social networks and music listening behavior.

To help investigate RQ2, we have constructed the following specific hypotheses:

H1: The geographical distance between countries reflects the difference in

music preference between countries.

H2: The distance in terms of wealth between countries is related to their distance in terms of music preference.

H3: Cross-country distance of the cultural dimension is related to cross-country differences in music preference.

H4: The linguistic distance between countries is related to cross-country differences in music preference.

H5: The density of friendship connections between countries is related to cross-country differences in music preference.

## Data and methodology

### LFM-1b dataset and data processing

The LFM-1b (www.cp.jku.at/datasets/LFM-1b) dataset is used in this study. With more than one billion music listening events created by 120,322 users, the dataset allows a large-scale analysis of music listening behaviors. The data acquisition procedure of LFM-1b is described in detail in a previous study [27]. The dataset covers the listening events of Last.fm users over nearly a decade (between 2005 and 2014). Users in the dataset originated from 208 countries, covering 3,190,371 artists. Nearly 54.13% of the users in LFM-1b dataset report their nationality. The distribution of users across the sampled countries and the populations of the country is shown in S1 Table. The number of users across countries is very unbalanced and to avoid unreliable results, we only retained countries with more than 1% of the total users in LFM-1b. This filtering resulted in 20 countries with 46,619 users and 678,640,512 listening events that cover 11,165,177 unique albums, 2,259,103 unique artists and 20 unique genres. The 20 countries are (in descendent order of user numbers): the United States (US), Russia (RU), Germany (DE), the United Kingdom (UK), Poland (PL), Brazil (BR), Finland (FI), Netherlands (NL), Spain (ES), Sweden (SE), Ukraine (UA), Canada (CA), France (FR), Australia (AU), Italy (IT), Japan (JP), Norway (NO), Mexico (MX), Czech Republic (CZ), and Belarus (BY). The genre labels are artist-based and correspond to those in Allmusic, a major online music repository [76].

To investigate cross-country friendship connections of the users in the dataset, we collected country information of their Last.fm friends by means of the user.getFriends API provided by Last.fm. According to Last.fm, *friends* of a user include both the *followees* and *followers* [77]. For the users in the 20 included countries, 40,076 unique friendship connections were identified, i.e., connections for which both connected users are included in the filtered LFM-1b dataset.

Using a large-scale dataset such as LFM-1b is of great significance. False positives and false negatives are two errors that often exist. Most measures or strategies to reduce one kind of error are often at the cost of increasing the likelihood of the other kind of error [78]. As Huron points out, using a large-scale dataset is the best way to minimize these two kinds of error [79]. The LFM-1b dataset was randomly sampled from all users on last.fm [27], and thus it does not suffer the problem of sampling bias [80]. Therefore, using the LFM-1b dataset contributes to mitigating two types of errors and thus improves the reliability of the results.

### Modeling cross-country differences in music preference

The between-country music preference distances are the dependent variables in this study, determined by the differences in albums, artists, and genres listening frequencies among countries.

We use album, artist, and genre listening frequencies to measure music preferences for multiple considerations. Album, artist, and genre are basic musical metadata types and are frequently involved in music retrieval and recommendation [81, 82], and as criteria for searching and browsing [83]. According to a survey[84], these three musical metadata types are important factors for both creating playlist and managing music collections. In the existing literature, album [85, 86], artist [23, 60, 87–89], and genre listening [2, 23, 87, 90] are often used to measure users’ music preferences[8]. Therefore, incorporating album, artist, and genre is not only for providing a relatively comprehensive picture that reflects users’ music preferences, but also for the consideration of the setting of the current music retrieval and recommendation systems. Besides, listening histories represented by album, artist, and genre may convey different and complementary information. Album and artist can inherently reveal information regarding cultural or country-specific aspects, e.g., the country of albums’ publishers, the ethnicity, language, and country of origin of artists. Genre preference is often considered related to users’ cognitive aspects, such as emotion [91], personality traits [46], and age [92]. Although genre can also uncover some cultural-specific information (for example, some genres, i.e., hip pop and rap, are closely related to black popular culture), it is often indirect. The average percentage of genre listening counts to the total listening frequencies in each country is shown in S1 Fig. It indicates that generally, the distributions of genres in most countries are similar to one another, which may imply that the between-country difference in genre listening is small.

Cosine similarity is widely used to measure proximity between two vectors, which is scale-invariant and especially efficient for sparse vectors since only the non-zero dimensions need to be considered [93]. Hence, based on the listening events in LFM-1b, we will calculate the cosine distances of album, artist, and genre preferences among countries, considering the invariance of cosine distance to the population size of countries and the inherent sparsity of the data (e.g., albums and artists). Specifically, each country is represented by a vector of albums, artists, or genres, with each dimension of the vector being the listening counts of the album, artist, or genre contributed by all the users in each country. For example, let C be a row vector with 20 dimensions where each dimension indicates a country, and let A be a column vector with 15,956,004 dimensions where each dimension represents an album. The value of the cell corresponding to Country C and Album A corresponds to how many times users in country C listened to Album A. Following the same strategy, we will construct the vectors of the artist and genre listening counts of users for all the sampled countries. Based on the vectors, the cosine distances of albums, artists, and genres between each pair of countries will be calculated.

### Modeling the cultural-socio-economic distances between countries

In this study, the cultural-socio-economic distance between countries is reflected by the following aspects: geographic, economic, linguistic, cultural distance, and the density of friendship connections among countries.

Based on the latitudes and longitudes of the capital cities of the countries, the *geographic distances* between countries can be calculated using Vincenty’s equations [94] which is based on the length of the shortest curve between two points on a spherical surface. This formula is widely used in geodesy and is accurate to within 0.5mm on the spheroidal earth and as such is considered sufficiently accurate for the purposes of this study [95]. In this study, we used the geodist package in Stata [96] to calculate the between-country geographic distance.

We define the *economic distance* between two countries as the between-country difference in the GDP per capita based on purchasing power parity (PPP), obtained from the World Bank (http://databank.worldbank.org/data/home.aspx) database. GDP per capita (PPP) is a better indicator than GDP (nominal) per capita for the purposes of this study as it can reflect the relative costs of living and the standard of living in countries rather than the whole country’s wealth. Based on the GDP per capita (PPP, current international dollars) of 20 countries from 2000 to 2017, we obtained the average GDP of each country. The average GDP ranged from 7,103 to 53,827 among these 20 countries, with NO and UA being the highest and lowest respectively.

For the *linguistic distance* between countries, the main language of a country is defined as the one spoken by the largest proportion of the population in that country which is obtained from the website of the Central Intelligence Agency (https://www.cia.gov/library/publications/the-world-factbook/fields/2098.html). Ethnologue (https://www.ethnologue.com/) provides the information of the global language family tree, from which we calculate the distance between two languages using the method proposed by Fearon [97] and Laitin [98]. This method has two main advantages over other methods (e.g., lexicostatistical distances between languages): it considers various aspects of languages’ characteristics, such as lexicon, syntax, and grammar; and it is available for almost all languages in the world [99]. Due to these merits, this method was later adopted by many researchers [100, 101]. According to Fearon and Laitin, the relative distance between languages depends on how many nodes they have in common in the language family tree. For example, English is classified as *Indo-European*, *Germanic*, *West*, *English;* whereas Swedish is classified as *Indo-European*, *Germanic*, *North Germanic*, *East Scandinavian*, *Continental Scandinavian*, *Swedish*, while Japanese is categorized into the branch of *Japonic*. The distance between English and Swedish is smaller than that between English and Japanese in that English and Swedish share two common nodes, while there is no common node for English and Japanese.

The *cultural distance* among countries is built on Hofstede’s cultural dimensions theory where national cultures are captured by the following six dimensions [43], each in a scale of [0, 120]:

Power distance (PDI) is the extent to which the less powerful individuals in a society accept and expect that power is distributed unequally. In countries high in power distance, a hierarchical order is clearly established, whereas in a low power distance society, people attempt to equalise the distribution of power;

Individualism (IDV) refers to the degree of preference for a loosely- or tightly-knit social framework. In highly individualistic societies, individuals often only relate themselves to their immediate family, whereas low individualism represents a society where tightly-integrated links extend beyond families;

Masculinity (MAS) indicates the extent to which the use of force in socially endorsed. Compared to feminine societies, in masculine societies designated gender roles are more expected (this definition of masculinity is on Hofstede’s website: https://geerthofstede.com/culture-geert-hofstede-gert-jan-hofstede/6d-model-of-national-culture/);

Uncertainty avoidance (UAI) refers to the attitude of individuals towards uncertainty and ambiguity. Societies scoring high in this index are intolerant of unorthodox behaviour and ideas;

Long-term orientation (LTO) is the tendency of connecting the past with the present and future actions/challenges. Countries with high scores in this index value adaptation and circumstantial, pragmatic problem-solving, rather than traditions and steadfastness;

Finally, Indulgence (IND) refers to the degree of gratification of enjoyment. Countries scoring high in this dimension tend to allow gratification of human desires concerning enjoying life and having fun. In countries with low Indulgence, gratification of needs is controlled and regulated by strict social norms.

The scores of countries in the six cultural dimensions were acquired from the homepage of Hofstede (http://geerthofstede.com/research-and-vsm/dimension-data-matrix/). We regard the difference in scores of each cultural dimension as the between-country cultural distance. The values of the six cultural dimensions for the sampled countries are shown in S2 Table.

The average *density of friendship connections* between country *i* and *j* is defined by Eq 1.

$$D_{i,j}^{friend}=\frac{1}{2}∙\frac{C_{ij}^{2}}{N_{i}\times N_{j}}$$ (1)

where *N_i_* and *N_j_* denote the total number of friendship connections generated by users in country *i* and country *j* respectively; *C_ij_* represents the friendship connections between country *i* and country *j*. Therefore, density *D_ij_* denotes the extent to which the listeners in any two countries are *friends* of each other.

An overview of the socio-cultural-economic aspects investigated in this study is presented in S3 Table.

### Cross-country relationship between differences in music preferences and cultural-socio-economic factors

In this study, we apply the Quadratic Assignment Procedure (QAP) [102, 103] via Double Dekker Semi-partialling [104] to examine whether the between-country differences in album, artist and genre preferences are associated with the differences in geographic, economic, cultural, linguistic proximities, and friendship connections between countries. In this study, each observation is a pair of countries and thus observations are interdependent. Ordinary Least Square regression analysis that assumes independent distribution of data may therefore lead to biased estimators [105–107]. In contrast, QAP explicitly takes into account the autocorrelation of errors in the dyadic dataset, and has been frequently used to address this problem [102, 108–111].

In our analysis, the explanatory variables are matrices of between-country differences in six cultural dimensions (i.e. PDI, IDV, MAS, UAI, LTO and IND), between-country density of friendship connections (FRIEN), geographical (GEO), economic (ECO), and language (LAN) distance among countries. The dependent variables are matrices of between-country differences in album, artist, and genre preferences which are included in Model 1 (Album), Model 2 (Artist) and Model 3 (Genre), respectively. To test the hypotheses, we will use UCINet, a software tool specialized for network analysis [112], to conduct multiple regression QAP by importing the 10 matrices of explanatory variables, and the matrix of dependent variables.

## Results and discussion

To answer RQ1 (cf. Section 3), we present the results and discussions of our analysis of country *differences in music preferences* and *in cultural-socio-economic factors*. Subsequently, we reveal the results of our QAP correlation and regression analysis to learn about the *relationship between the differences* in music preferences and cultural-socio-economic factors (RQ2).

To avoid the bias resulting from the unbalanced distribution of listening counts across countries, we look into both the raw listening counts and proportions of listening counts by normalizing albums, artists, and genres listening frequencies for each country. As the results are very similar, we have only reported results on the raw listening counts in this section.

### Cross-country differences in music preference and cultural-socio-economic factors

**Fig 2:**
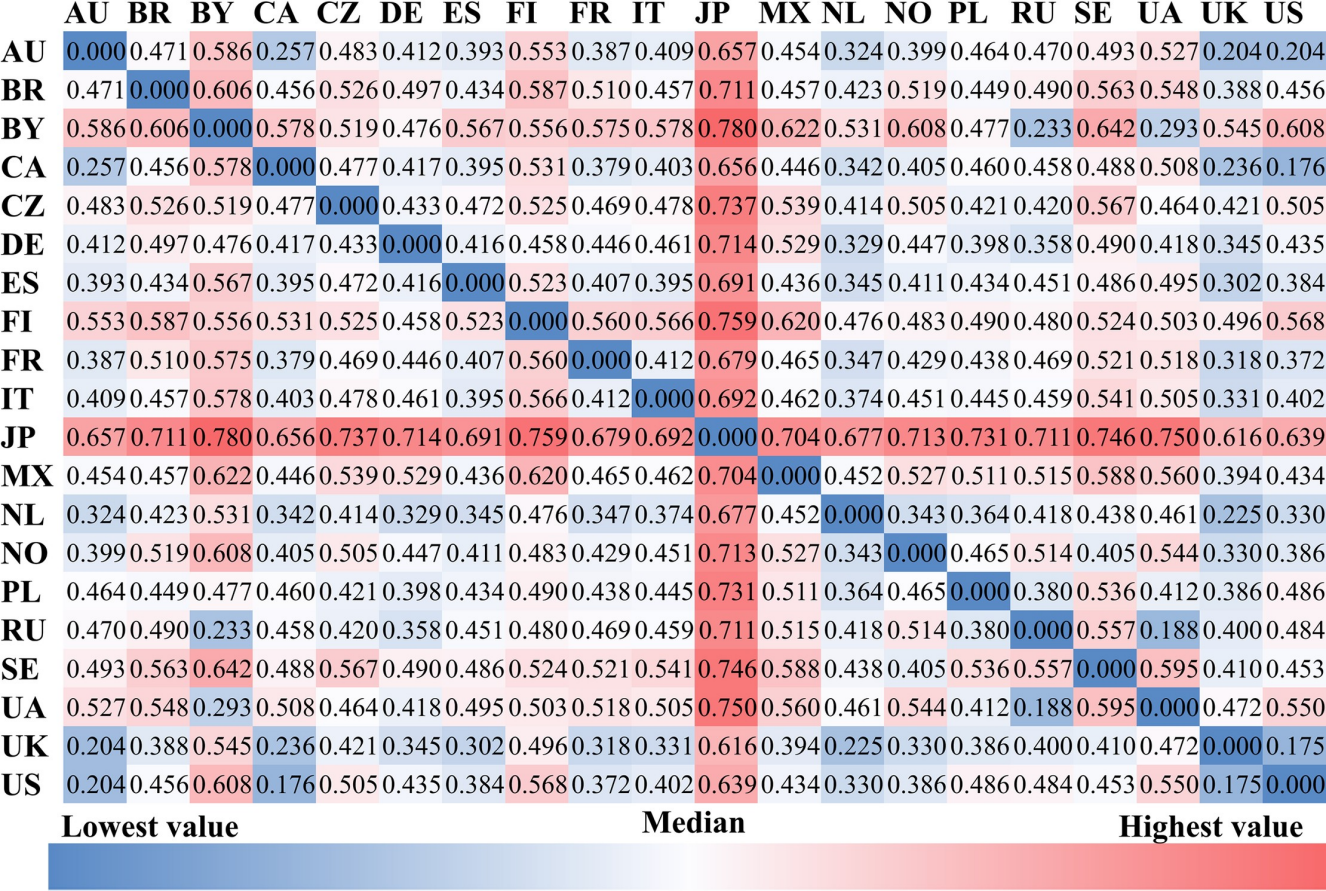
The cosine distance of album preferences among countries.

**Fig 3:**
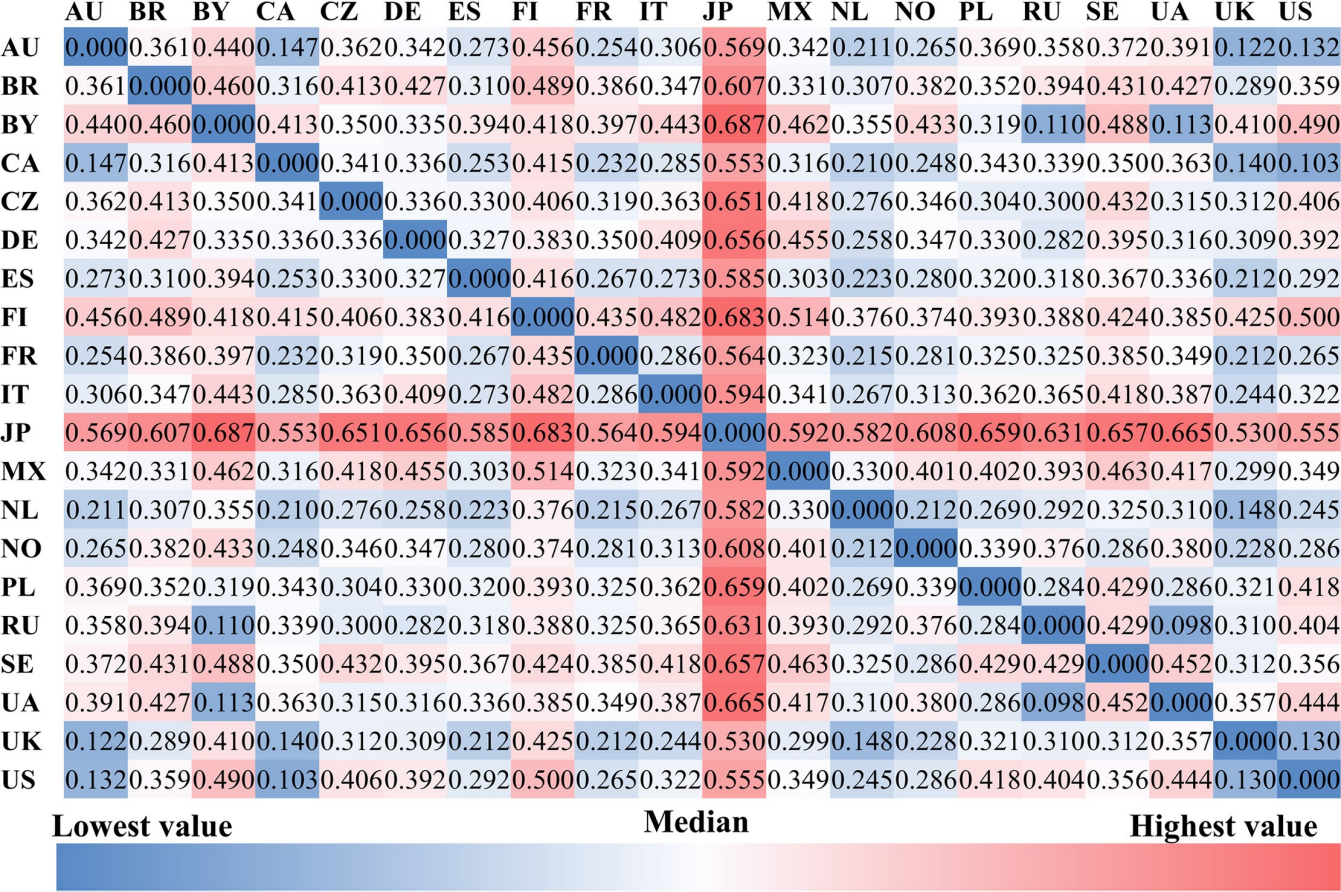
The cosine distance of artist preferences among countries.

**Fig 4:**
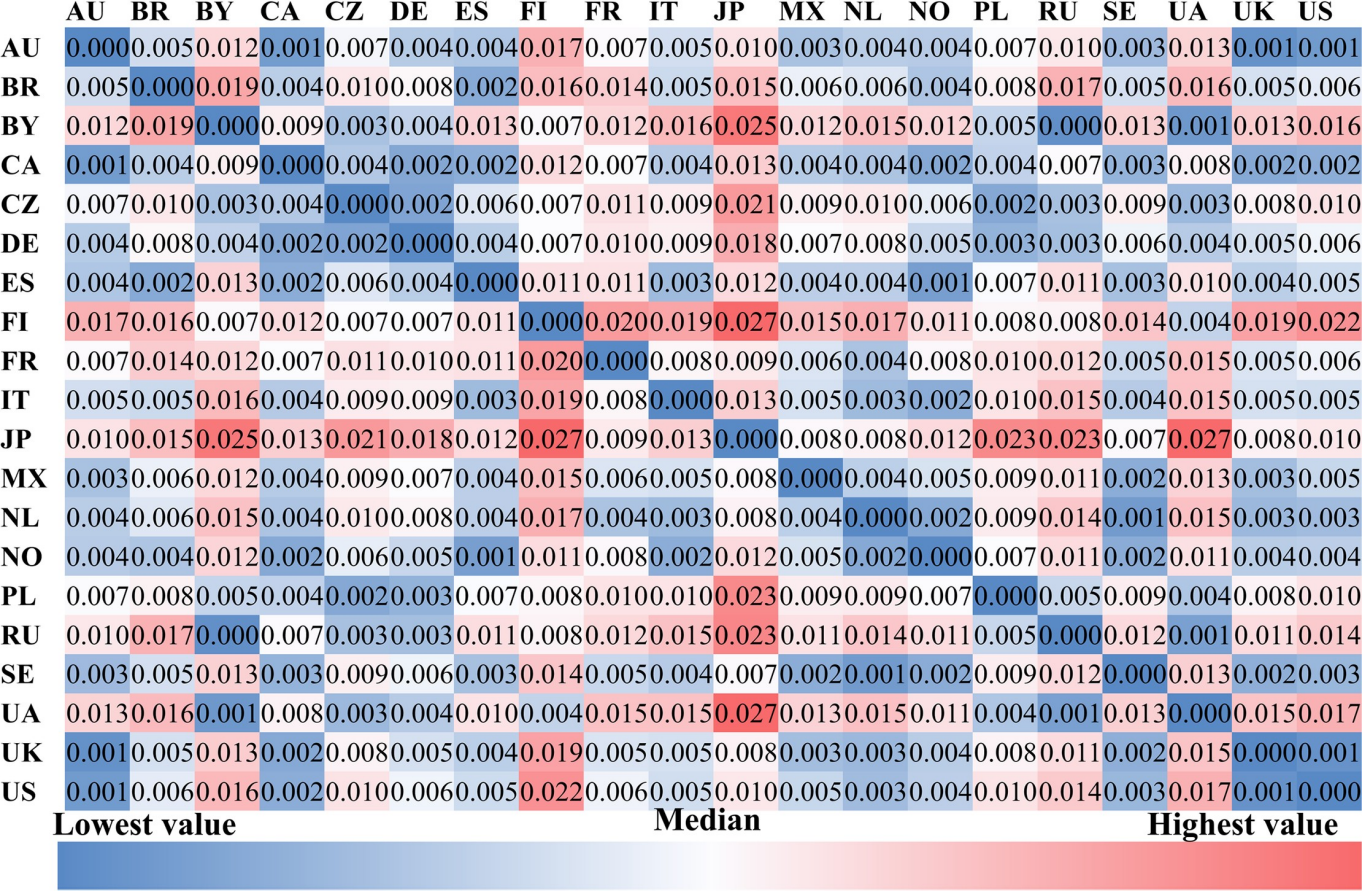
The cosine distance of genre preferences among countries.

Figs 2 to 4 present the between-country (cosine) distance of *music preferences*, for albums, artists, and genres, respectively. Shades of blue indicate smaller distance, shades of red indicate larger a distance between the respective pairs of countries. The most salient characteristic is that the distances between Japan and all other countries are larger than those between all other pairs of countries, irrespective of the level at which music preference is modeled (album, artist, or genre). Furthermore, the average cosine distance of album preferences across all country pairs (0.47) is larger than that of artist (0.36) and genre preferences (0.01). Also in common across the three figures are: 1) the distances between the U.S., U.K., Canada, and Australia are shorter; 2) the distances between Russian, Ukraine, and Belarus are shorter; and 3) the distances between Finland, Sweden, and Norway are not short. The first two observations reflect cultural similarity and/or historical connections, while the third appears to contradict with the geographic proximity of the three Nordic countries.

**Fig 5:**
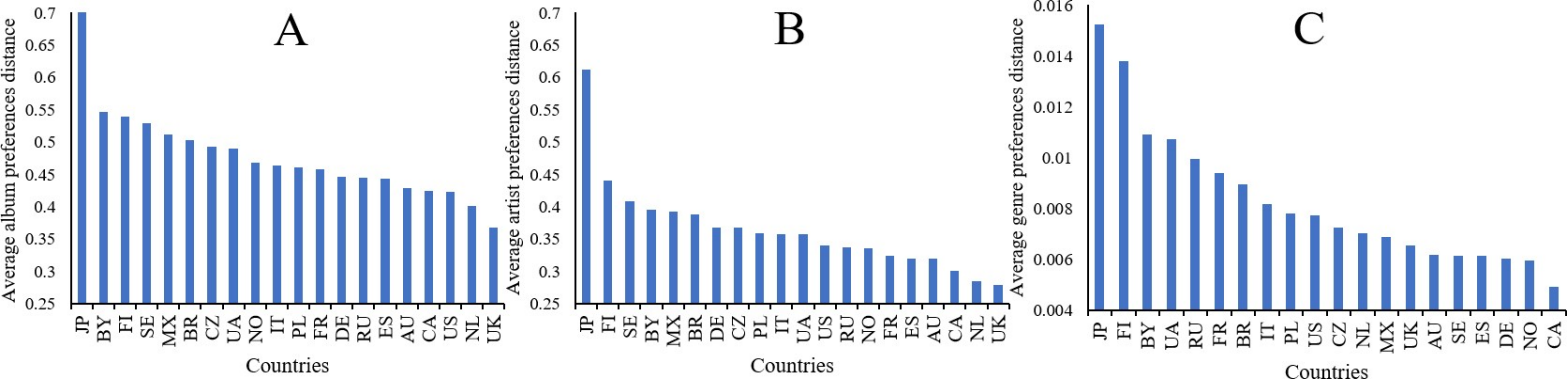
The average between-country cosine distances of music preferences. (A): distances of album preferences; (B): distances of artist preferences; (C): distances of genre preferences.

Fig 5 shows the average between-country distance of album, artist, and genre preferences of each country. The average between-country distance of the U.K., Canada, the Netherlands, and Norway is smaller than that between other countries, whereas Japan, Belarus, Finland, and Sweden have larger average between-country distances than the remaining countries.

The between-country differences in genre listening preferences are not as pronounced as those in album and artist preferences. Taking the inequality or diversity of album, artist, and genre listening across countries as an example by means of a Lorenz curve (compared to other techniques, e.g., Gini index, Lorenz curve provides a graphical representation of the distribution of music listening, which facilitates our understanding of the inequality of music listening across countries) [113], we found that genre listening is more balanced than that of albums and artists as reported in S2 to S4 Figs in Supporting Information. Specifically, 90% of genres contributed more than 60% of the total listening counts in all countries. However, 90% of artists and albums listened to by users in most countries have approximately 10% and 20% of the total listening frequency, respectively, in each country. The Lorenz curves suggest that the difference in genre listening is far slighter than that in artist and album listening.

**Fig 6:**
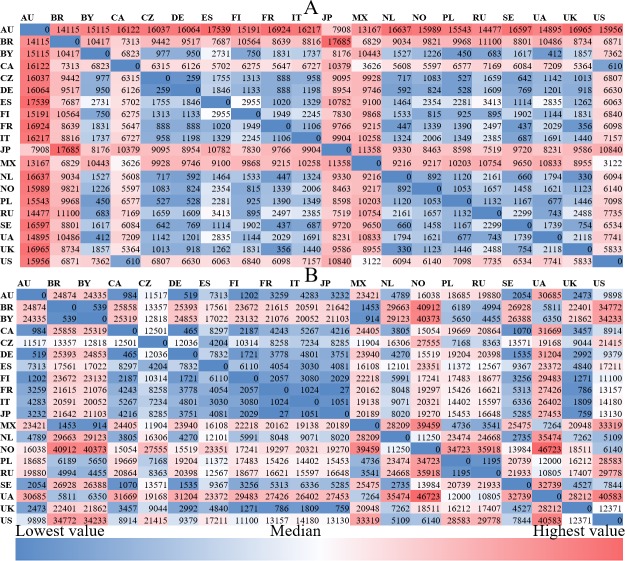
The between-country geographical and economic distances. (a): geographical distances in kilometers; (B): economic distances in current international dollars.

**Fig 7:**
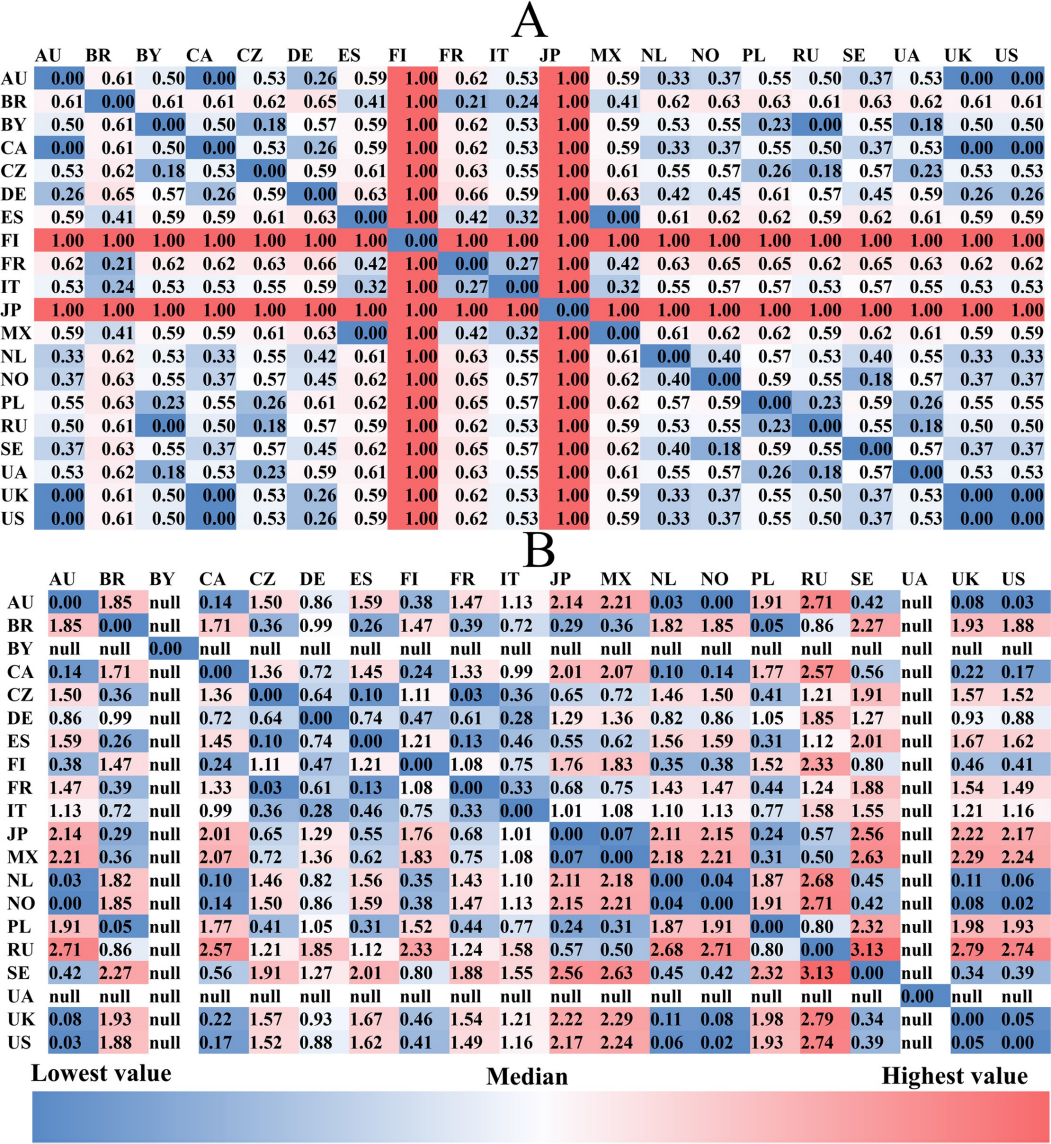
The between-country linguistic and cultural distances. **(a): linguistic distances; (b): cultural distances**; “null” for UA and BY is due to the missing value in the original culture scores of these countries released by Hofstede (http://geerthofstede.com/research-and-vsm/dimension-data-matrix/). Although cultural scores for BY and UA are unavailable, other aspects of these two countries have values (e.g., geographic, economic and linguistic aspects, and friendship connections) which are included in the QAP regressions.

**Fig 8:**
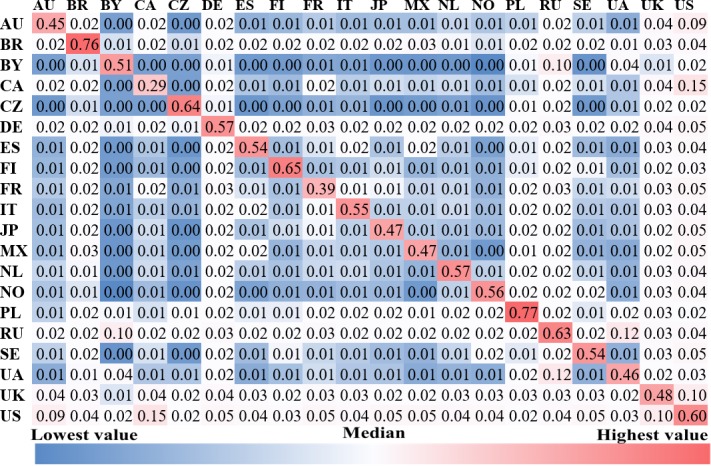
The density of friendship connections among countries.

Figs 6 to 8 illustrate the *socio-economic distance* among countries from the aforementioned five cultural-socio-economic dimensions. As shown in Fig 6, Australia is unsurprisingly the country which is geographically furthest away from all other countries. In the matrix of GDP distance among countries presented in Fig 6. The average GDP ranged from 7,103 to 53,827 among these 20 countries, with NO and UA being the highest and lowest respectively.

As for *language differences*, the main languages in Japan and Finland are considerably distinct from those in other countries as shown in Fig 7(A). This is consistent with the fact that Japanese and Finnish are assigned to Japonic and Uralic language families respectively, while the main languages in the remaining 18 countries are all included in the Indo-European language family.

To investigate *cultural distances*, we performed a Principal Component Analysis on the six cultural dimensions (rather than the average distances as the six dimensions may not be orthogonal to one another), to obtain an overall culture score for each country, for illustrative purposes. As can be seen in Fig 7(B), Russia and Sweden have a greater disparity in culture from the remaining countries.

Fig 8 reports the *density of friendship connections* among countries. It is not surprising that most of the users’ *friends* come from their own countries. The color of cells in the diagonal is far darker red than that of the remaining cells, especially for Poland, Brazil, Finland, the Czech Republic, and Russia. This suggests that users in these five countries are more inclined to maintain friendship with those who originate from the same country. However, Canadian (0.29) and French (0.39) users show a lower density of friendship connections with other users from the same country, implying that they have a more open and tolerant attitude towards following foreigners, or are frequently followed by users in other countries. In addition, there are strong friendship connections between some country pairs, e.g., the U.S. and Australia, Russia and Belarus, the US and Canada, Ukraine and Russia, and the U.K. and the U.S. Intuitively, these country pairs are either geographically adjacent or culturally close to each other.

Most of the country pairs show differences in album and artist preferences, while half of the country pairs show similar genre preferences. Japan and Finland have larger distances of music preferences to other countries, which may be related to the fact that the Japanese and Finnish languages are of very different origins from the major languages in the other 18 countries. Users tend to keep friendship connections with those from the same country except for users in Canada and France. Additionally, some country pairs that are either geographically or culturally close to each other show intensive friendship connections, e.g., the U.S. and Australia, Russia and Belarus, the U.S. and Canada, Ukraine and Russia, and the U.K. and the U.S.

### Cross-country correlation between differences in music preferences and cultural-socio-economic factors

To test the hypotheses listed in Section 3, we create and compare three models to explore the relationship between cross-country distances in music preference (as represented by album, artist, and genre listening frequencies) and cross-country distances in geographical, economic, language, cultural and friendship density aspects. It should be noted that the reason why we used six cultural distances rather than the overall cultural distance is that we specifically sought to discover which dimensions of the six cultural factors account for music preference distances across countries. The QAP correlation coefficients among variables are reported in Table 1, showing a high correlation of between-country distance in album, artist, and genre listening frequencies. To avoid imprecise estimations caused by multicollinearity of the explanatory variables, we calculated the mean variance inflation factor score (1.79), which is far lower than critical point 10 [114], implying that multicollinearity can be ignored in this study. The QAP regression results are presented in Table 2.

**Table 1:** The QAP correlation results.

	ALBUM	ARTIST	GENRE	GEO	ECO	LAN	FRIEN	PDI	IDV	MAS	UAI	LTO
**ARTIST**	0.971***	1										
**GENRE**	0.692***	0.706***	1									
**GEO**	0.217	0.248	0.087	1								
**ECO**	0.184	0.117	0.121	-0.043	1							
**LAN**	0.714***	0.745***	0.600	0.066	0.046	1						
**FRIEN**	-0.537***	-0.430***	-0.272***	-0.062	-0.135**	-0.425***	1					
**PDI**	0.183	0.149	0.092	-0.034	0.570***	0.211	-0.160*	1				
**IDV**	0.217	0.215	0.090	0.354*	0.483**	0.136	-0.052	0.458**	1			
**MAS**	0.377**	0.34*	0.168	-0.056	0.003	0.317*	-0.231*	0.005	-0.106	1		
**UAI**	0.141	0.144	-0.014	-0.101	0.264*	0.241*	-0.116	0.566**	0.266*	0.120	1	
**LTO**	0.275**	0.267**	0.157*	0.266**	0.034	0.081	-0.177*	0.019	0.112	0.010	0.025	1
**IND**	0.328**	0.269*	0.225*	0.112	0.329**	0.140	-0.334***	0.416**	0.326**	-0.037	0.398**	0.346**

Notes: Significance levels

***: p < 0.001

**: p < 0.01

*: p < 0.05.

**Table 2:** The QAP regression results.

Variable	Model 1	Model 2	Model 3
	(Album)	(Artist)	(Genre)
GEO	0.119	0.145	0.010
ECO	0.124	0.071	0.100
LAN	0.643***	0.688***	0.626***
FRIEN	0.000	-0.001***	-0.001***
PDI	-0.033	-0.055	-0.016
IDV	0.015	0.036	-0.041
MAS	0.170**	0.126*	-0.016
UAI	-0.107	-0.065	-0.209
LTO	0.121**	0.124**	0.050
IND	0.193*	0.126*	0.175
Adjusted R2	0.635***	0.645***	0.418***
N of Obs	380	380	380

Notes: All coefficients presented are standardized coefficients

Significance levels

***: p < 0.001

**: p < 0.01

*: p < 0.05.

In Table 2, the adjusted *R*^2^ in all models are significant, indicating that the between-country distances in the geographic, economic, linguistic, cultural, and the friendship density aspects together are responsible for nearly 62.8%, 64.2% and 41.3% of the variance in the matrix of the album, artist and genre preference distances among countries, respectively.

All models revealed no significant association between the geographical and economic distance on album, artist, and genre preferences’ distance at the country level. Accordingly, hypotheses 1 and 2 are not supported in all models. This result may be accounted for by the fact that only considering the geographical distance between countries based on the pure coordinates does not reflect their difference in music preferences. As for economic distance, although at the individual level it was confirmed that music preference varies by income, this was however not reflected at the country level. As Levine [38] emphasized, the correlation between specific cultural behaviors and social status strongly depends on the social context [38]. At the country level, there might be very different social contexts, and thus the income difference among countries cannot explain the difference in music preference.

The distances of three cultural dimensions among countries show significantly positive effects on both the album and artist preferences distances among countries. In model 1, the coefficient for masculinity (β = 0.17; p<0.01), long-term orientation (β = 0.12; p<0.01) and indulgence (β = 0.19; p<0.05) is positive and significant. One standard deviation increase in the cross-country differences in masculinity, long-term orientation and indulgence, is related to 0.17, 0.12, 0.19 standard deviation increases in the cross-country differences in album preferences, respectively. Masculinity (β = 0.13; p<0.05), long-term orientation (β = 0.12; p<0.01) and indulgence (β = 0.13; p<0.05) are also positively associated with the artist preferences distance as reported in model 2. This indicates that one standard deviation increase in the cross-country differences in masculinity, long-term orientation and indulgence, is related to 0.13, 0.12, 0.13 standard deviation increases in the cross-country differences in album preferences, respectively. However, it seems that there is no significant association between the cross-country differences in cultural aspects and those in genre preferences. Therefore, hypothesis 3 is partially accepted.

In the six cultural dimensions, between-country distances of *masculinity*, *long-term orientation* and *indulgence* are positively related to both the album and artist preference distances among countries. First, *masculinity* culture clearly differentiates gender roles which are related to the expression and perception of emotion [115]. It has been widely agreed that music listening preference and emotions are strongly connected to each other [116, 117]. Some literature has also demonstrated that *masculinity* can explain the gender difference in personality traits [118] which are empirically connected to music behavior studies [119, 120]. Consequently, it is possible that on the country level, the music listening differences account for the *masculinity* differences that lead to gender difference in both emotion and personality traits that impact music behaviors. Second, some studies have offered evidence that people in countries with low *long-term orientation* have less preference for diverse artists since they honor traditions and value steadfastness [23], and thus may rather listen to more traditional music. Furthermore, a recent study found that individuals in countries that score high in *long-term orientation* tend to be more patient [121] which might impact their music preferences. This assumption will be tested further as part of future work. Third, in countries scoring high on *indulgence*, people have more freedom to control their daily life and choose the way in which they enjoy life and have fun. Given that listening to music is an important entertainment activity, the difference in *indulgence* can bring about the difference in music preference across countries. However, it seems that there is no significant association between the cross-country differences in cultural aspects and those in genre preferences. As shown in Fig 5 and S2 to S4 Figs, the between-country differences in genre listening are not as pronounced as those in album and artist listening, which may be a reason for the result that the cross-country differences in some cultural aspects (MAS, LTO, IND) are significantly correlated to the cross-country differences in *album* and *artists* preferences, but are not significantly related to the cross-country differences in *genre* preferences. Furthermore, as compared to albums and artists, genre can only provide limited information to users, such as emotion and music style, and may not tell more about country-specific or culture-specific information.

The between-country distance in main languages has a positive association with the album, artist, and genre preferences distance among countries. The coefficient for the linguistic distance between countries is significant and positive in all models (β = 0.63–0.69; p<0.001). The cross-country linguistic distance seems the most important factor for predicting the album, artist and genre preferences distances among countries since the standardized coefficient for this variable is larger than those for other variables in all models. One standard deviation increase in the cross-country linguistic distance leads to 0.64, 0.69 and 0.63 standard deviation increases in the album, artist and genre preferences differences among countries. Hence, hypothesis 4 is supported. One possible explanation is that familiarity is a central factor influencing music preference [122, 123]. It not only refers to the assumption of having heard the song before, but also can be reflected by familiarity degree of language in songs [44]. Hence, listeners may prefer listening to songs in languages with which they are familiar.

There is a significantly negative relationship between the density of friendship connections among countries, and preference distance among countries measured by artist and genre. As displayed in models 2 and 3, a higher density of friendship connections among countries indicates less difference in their artist and genre preferences. One standard deviation increase in the density of friendship connections among countries is related to 0.001 standard deviation decrease in the cross-country differences in artist and genre listening. In contrast, this relationship does not exist when album listening preference is the independent variable and thus hypothesis 5 is partly accepted. It is well understood since people’s behaviors and friendship interact [124]. Specifically, users with similar music taste incline to *follow* and be *followed* by each other. On the other hand, the user’s music taste or listening behavior may also be influenced by his or her *followings*. The relationship between the density of friendship connections and the differences in music preferences is arguably reciprocal: the difference of users in music preferences may influence their friendship connections, and in turn friendship connections also possibly shape their music taste. Whether the music preferences influence the friendship connection, or the latter influences the former, or both, needs to be investigated further in future research by means of econometric techniques, e.g., instrumental variable techniques and difference-in-differences models [125].

## Robustness check based on word vector technique

To mitigate the possible problem in measuring the between-country difference caused by the sparsity of the album and artist listening data, we used word vector [126], a popular word embedding technique in Natural Language Processing, to represent album and artists in low-dimensional dense vectors that capture the numerical features of albums/artists. It is noteworthy that the genre listening data for countries are not sparse since there are only 20 genres, and thus the word vector method is only applied to album and artist listening data.

The basic idea of a word vector is that words appearing in similar contexts tend to have similar meanings. The context of a word is represented by its neighboring words. Given a corpus, i.e., a set of sentences, the model loops on the words of each sentence and either uses the current word to predict its surrounding words (Skil-Gram model) or uses these surrounding words to predict the current word (Continuous Bag of Words model) [126]. The limit on the number of neighboring words is determined by “window size”. The weights that constitute a word vector are typically generated by estimating the probability that other words are contextually close to a given word based on the corpus.

We consider an album/artist ID as a word, the listening history (of album/artist) of a user as a “sentence”, and all the listening histories of sample users as a corpus. In the corpus, there are 11,165,177 unique albums and 2,259,103 unique artists. In the listening history of each user, the IDs of albums/artists are organized in chronological order according to the time when users listened to a certain album/artist. The order of album/artist IDs carries the information about the albums/artists users listened to before or after users listening to a specific album/artist. Using Skip-gram Word2vec with Negative Sampling [127, 128], we fed the listening history of each user into the model to obtain the word vector of albums/artists. The window size is set to 20, which means that 20 album/artist before and after a given album/artist would be included as neighboring album/artist. The dimensionality of the output word vectors is set to 100. Any album/artist appearing less than 5 times in the corpus is ignored by the model. This procedure reduces the final number of unique albums to 299,502, and that of unique artists to 287,353. Other parameters are set by default of the Word2vec module implemented in the Gensim library. The outputs are a 100ⅹ299,502 dimensional feature matrix of albums, and a 100ⅹ287,353 dimensional feature matrix of artists. 100-dimensional vectors of each album/artist capture the feature of them.

We calculate the between-country distance in album/artist listening based on the feature matrices of albums/artists. Taking album as an example, let *C* = {*c*_1_, …, *c*_20_} be the set of 20 countries, and *A =* {*a*_1_, …, *a*_299,502_} the set of unique albums users in 20 countries listened to and appearing in the feature matrix of albums. A country is represented as a 299,502 dimensional vector. The value of each dimension of the vector denotes the listening frequency of album *a∈A* users in country *c∈C* listened to. Then, we captured a 20 ⅹ 299,502 matrix that presents the listening frequency of albums users in 20 countries listened to. We multiplied this album listening matrix by the feature matrix of albums (299,502ⅹ100), then obtained a 20ⅹ100 dimensional matrix of countries that captures the sum of the features of albums to which users in a country listened, weighted by the listening frequency of each album to which users in the country listened. Based on this low-dimensional matrix where data sparsity is eliminated, we calculated the between-country cosine distance of countries in album listening. Following the similar procedure, the between-country cosine distance of countries in artist listening is obtained. S5 and S6 Figs show the heatmaps of the between-country cosine distance in album and artist listening based on word vector technique, respectively. Generally, the relative between-country distances in these two figures are similar to those in Figs 2 and 3 although the scale is different. For example, in S5 and S6 Figs, the distance in album and artist listening between JP and the remaining countries is larger than that between any other country pairs.

Using the between-country distance in album and artist listening based on word vector technique, we ran QAP regressions again. The results are shown in S4 Table. Generally, most of the results are consistent with the findings, despite the non-significant relationship between IND and the between-country distance in album listening, and the non-significant correlation between MAS and the between-country distance in artist listening.

## Conclusions and future work

Users’ contextual information, including geographical, economic, linguistic, cultural and social factors, play a critical role in music listening and preferences. Revealing the relationship between the difference among countries in music preference and in the aforementioned context categories is of vital importance to answer which cultural-socio-economic factors should be integrated into music retrieval and recommender systems, for instance, to provide a better personalization of results. Built on a theoretical framework developed from classic theories in cultural and social studies, and using Quadratic Assignment Procedure techniques, this study uncovered various between-country differences in cultural-socio-economic aspects and in album, artist, and genre listening preferences (RQ1), as well as in the relationship between these differences (RQ2). We found that the between-country differences in genre listening are much smaller than those in album and artist listening. We found no significant association between neither geographical nor economic distance and the music preferences’ distance at the country level, while distances on language, culture and friendship are significantly related to cross-country differences on music preferences. Future research includes taking into account music content and music context descriptors to model music preferences in multifaceted ways, to subsequently investigate whether there are more subtle differences (e.g., between cultural aspects of the listener and certain melodic or lyrical music features).

The findings are especially meaningful to mitigate the cold-start problem in cases when only the country information of a new user is available to the recommendation system. Traditionally, user-item recommendation requires obtaining a sufficient amount of listening records of users which needs time to accumulate. Furthermore, if there are only a small number of users in some certain countries, capturing the average music preferences of these countries is challenging. Based on the estimated coefficients of the between-country distances in various socio-cultural-economic variables in this study, the differences between any two countries in album/artist/genre listening can be estimated since socio-cultural-economic data of countries is publicly available. According to the estimated between-country differences in music listening, the recommendation system can recommend new users popular albums/artists/genres in the countries that have the smallest estimated differences in music listening with the countries of these users. Additionally, the contextual variables could be modeled as explicit dimensions in the user-item matrix used for factorization. Collaborative filtering approaches could be enhanced by context-based filtering of users according to cultural-socio-economic aspects. However, our findings show that not all contextual variables account for users’ music preferences. For example, the economic income, the geographic location, and some cultural aspects of users’ countries may not be incorporated. In contrast, we found that the cross-country distance in main languages has a positive association with the cross-country difference in album, artist and genre listening. The between-country difference in three cultural aspects (i.e., masculinity, long-term orientation, and indulgence) is positively related to both the album and artist listening distance across countries. Furthermore, the density of friendship connections among countries negatively relates to the cross-country distance regarding artist and genre listening. In addition, the algorithms for album/artist recommendation and those for genre recommendation should be designed differently. For example, the genre recommendation may not necessarily consider the cultural aspects of users’ countries.

There are some limitations to this study. We used cosine distance to measure the between-country distance in various socio-cultural-economic aspects and the between-country differences in album, artist, and genre listening preferences. Although cosine distance is invariant to scale, the sparsity of the data (e.g., album and artist listening) may increase the distance between countries, which could potentially affect the reliability of the measure of music preferences. However, compared to other similarity measures (e.g., Euclidean distance, Manhattan distance), cosine distance performs better with high dimensional and sparse data [93, 129, 130]. Furthermore, we used word2vec technique [128] to mitigate the possible problem caused by the sparsity of the album and artist listening data through a robustness check. The results indicate that the main findings are robust and reliable. Hence, this problem may not hamper the reliability of the findings. Second, the LFM-1b dataset extracted from Last.fm suffers the community bias, which means that it only contains listening behaviors of music listeners who used Last.fm or other online music services connected to Last.fm, such as Spotify and SoundCloud. However, this challenge also exists for data generated from any other online music platforms, since users’ listening behaviors captured by these platforms only reflect a part of the real-world situation. The LFM-1b dataset has advantages in its substantial size and a wide range of detailed user-specific information, compared to other publicly available datasets, e.g., the Million Song Dataset [131] and Celma’s dataset [132]. Due to the data availability, despite the community bias, we still consider LFM-1b as a trustful dataset, given the fact that it has emerged as an increasingly used dataset in MIR [16, 27, 133]. Besides, we measured users’ album/artist/genre listening based on their listening histories. While listening histories cannot tell whether all the songs were actually listened to by the user, or they were always actively selected by the users, it is largely safe to assume that in most cases the users did listen to the songs in the listening histories and they at least did not dislike these songs. Therefore, the results based on the aggregation of large-scale listening histories are valid in studying users' listening preferences. In addition, as the dataset contains only online music listening histories, presumably only listeners who were able to operate networked digital devices were included in the sample. Therefore, readers should be cautious when generalizing the results.

## Supporting information

S1 Fig The average percentage of genre listening counts to the total listening frequencies in each country. 1 to 20 in the first row denote the following genres: rnb, rap, electronic, rock, new age, classical, reggae, blues, country, world, folk, easy listening, jazz, vocal, children, punk, alternative, spoken word, pop, heavy metal.
(TIF)

S2 Fig Lorenz curves of album listening frequency. The y-axis indicates the percentage of the total listening frequencies and the y-axis indicates the percentage of albums.
(TIF)

S3 Fig Lorenz curves of artist listening frequency. The y-axis indicates the percentage of the total listening frequencies and the y-axis indicates the percentage of artists.
(TIF)

S4 Fig Lorenz curves of genre listening frequency. The y-axis indicates the percentage of the total listening frequencies and the y-axis indicates the percentage of genres.
(TIF)

S5 Fig The between-country distances in album listening based on word vector technique. (TIF)

S6 Fig The between-country distances in artist listening based on word vector technique. (TIF)

S1 Table The distribution of users across the sampled countries and the populations of the countries. (DOCX)

S2 Table The values of the six cultural dimensions for the sampled countries. (DOCX)

S3 Table Overview of socio-cultural-economic aspects investigated in this study. (DOCX)

S4 Table The QAP regression results for robustness check. (DOCX)

S1 Data The friendship connections of the sampled users. (XLSX)

S2 Data The latitudes and longitudes of the capital cities of the sampled countries. (XLSX)

S3 Data The classification of countries’ dominant languages. (XLSX)

S4 Data GDP per capita (PPP) of the sampled countries. (XLSX)
