# Correction: The relation of culture, socio-economics, and friendship to music preferences: A large-scale, cross-country study

**DOI:** 10.1371/journal.pone.0212495

**Published:** 2019-02-13

**Authors:** Meijun Liu, Xiao Hu, Markus Schedl

There is an error in the Corresponding Author’s email. The correct email is: xiaoxhu@hku.hk.

There in an error in Data and methodology section, under the sub-heading “Modeling cross-country differences in music preferences.” The fourth sentence of the last paragraph should read as follows: For example, let C be a row vector with 20 dimensions where each dimension indicates a country, and let A be a column vector with 11,165,177 dimensions where each dimension represents an album.
