# Supplementary figures and images for: The relation of culture, socio-economics, and friendship to music preferences: A large-scale, cross-country study
Source: PLoS One. 2018 Dec 14;13(12):e0208186. doi: 10.1371/journal.pone.0208186 (PMC6294554; doi:10.1371/journal.pone.0208186)

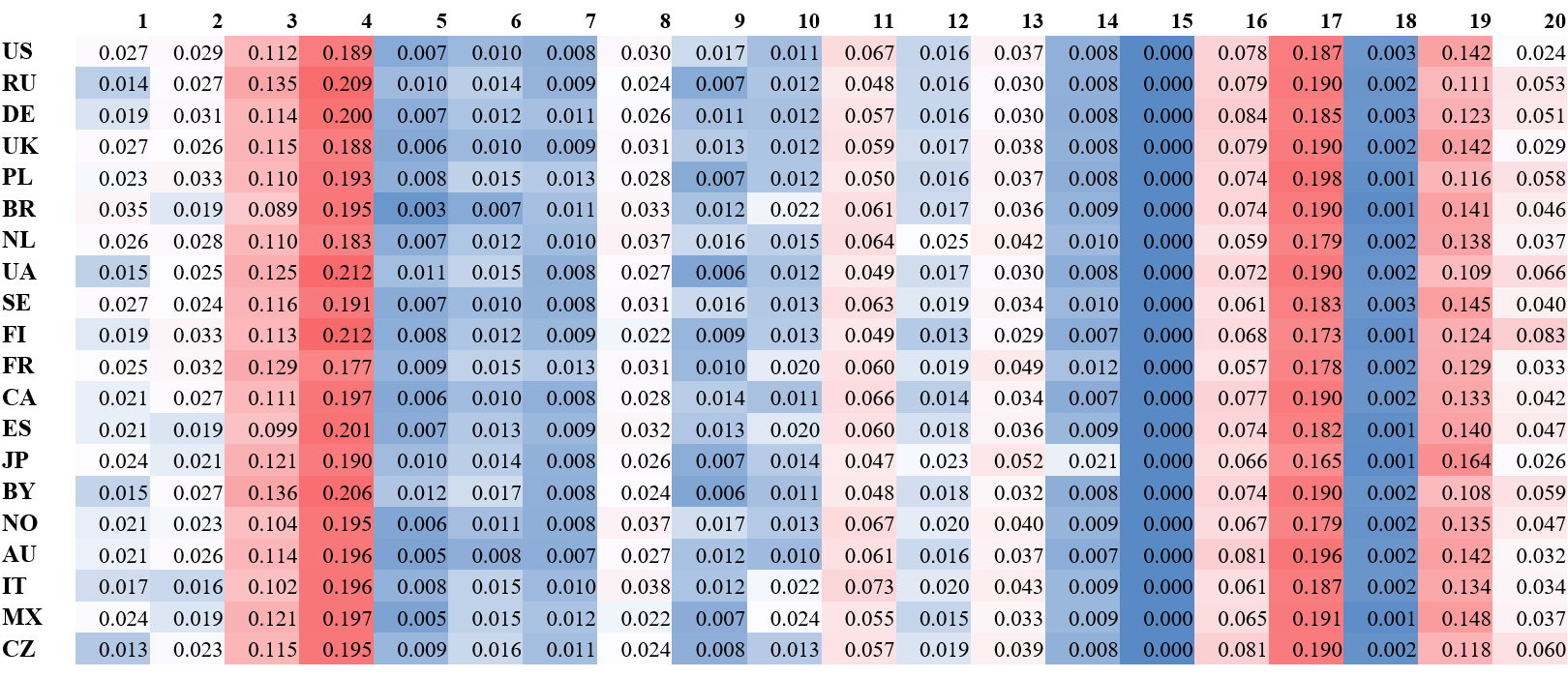

Supplement: S1 Fig — 1 to 20 in the first row denote the following genres: rnb, rap, electronic, rock, new age, classical, reggae, blues, country, world, folk, easy listening, jazz, vocal, children, punk, alternative, spoken word, pop, heavy metal. (TIF) [file pone.0208186.s001.tif]

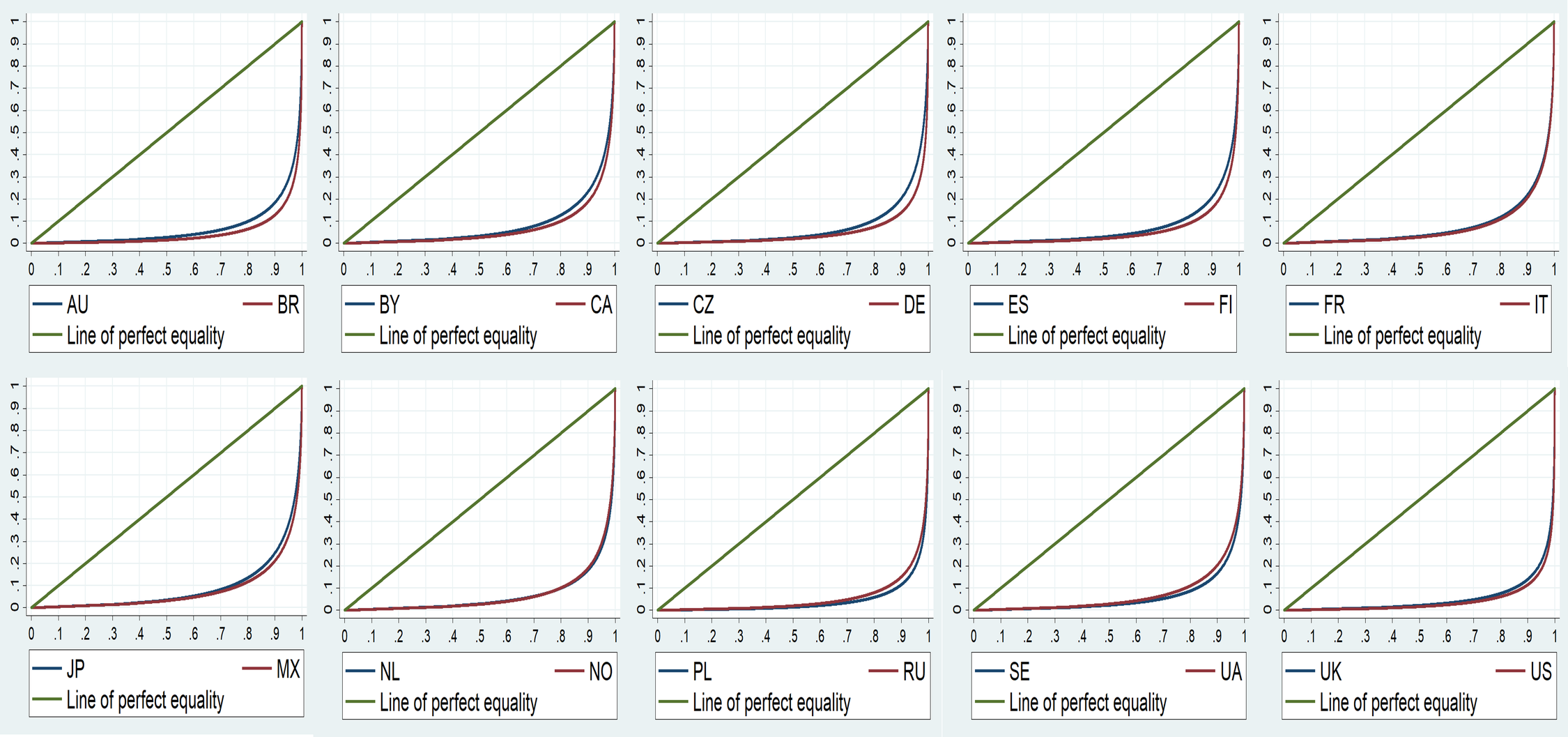

Supplement: S2 Fig — The y-axis indicates the percentage of the total listening frequencies and the y-axis indicates the percentage of albums. (TIF) [file pone.0208186.s002.tif]

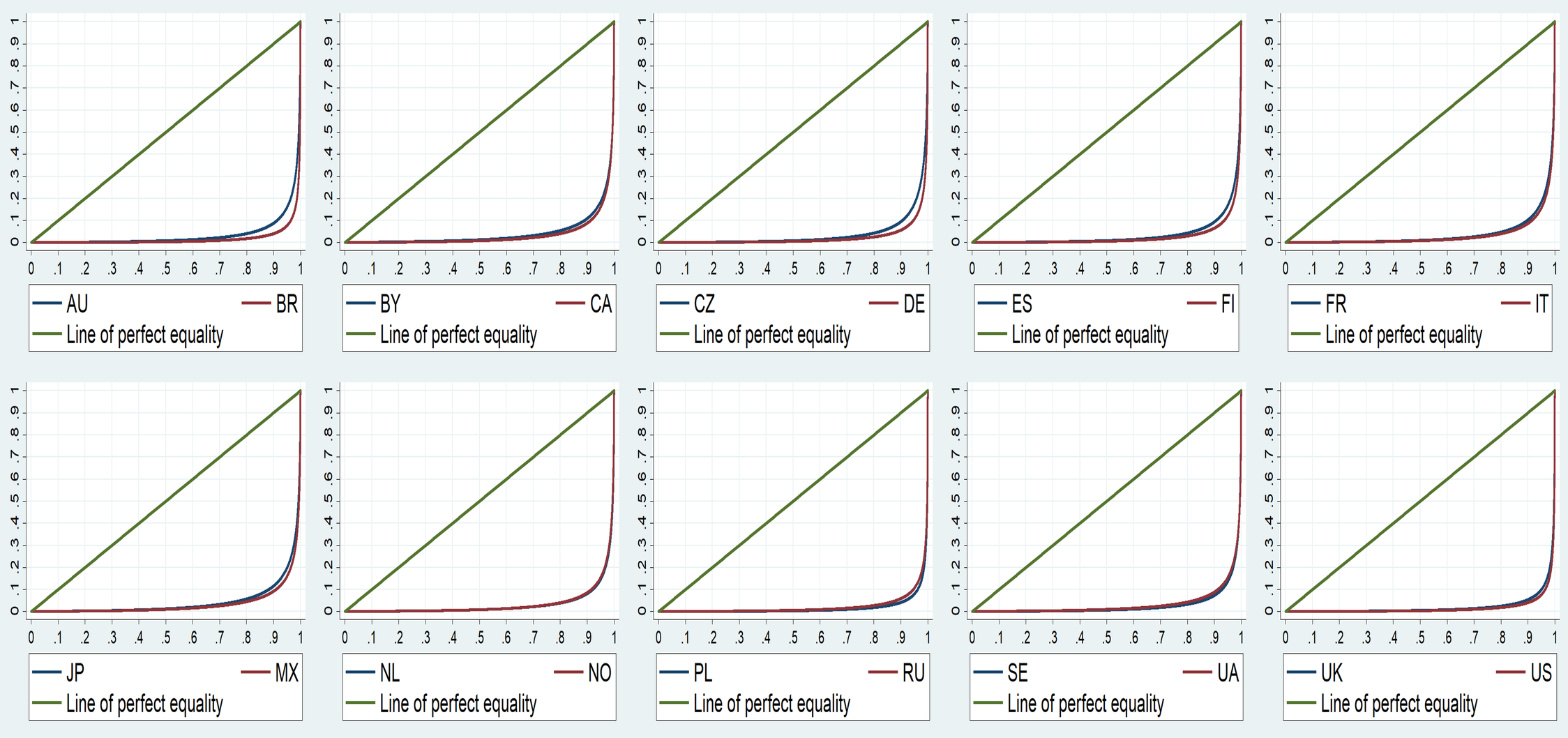

Supplement: S3 Fig — The y-axis indicates the percentage of the total listening frequencies and the y-axis indicates the percentage of artists. (TIF) [file pone.0208186.s003.tif]

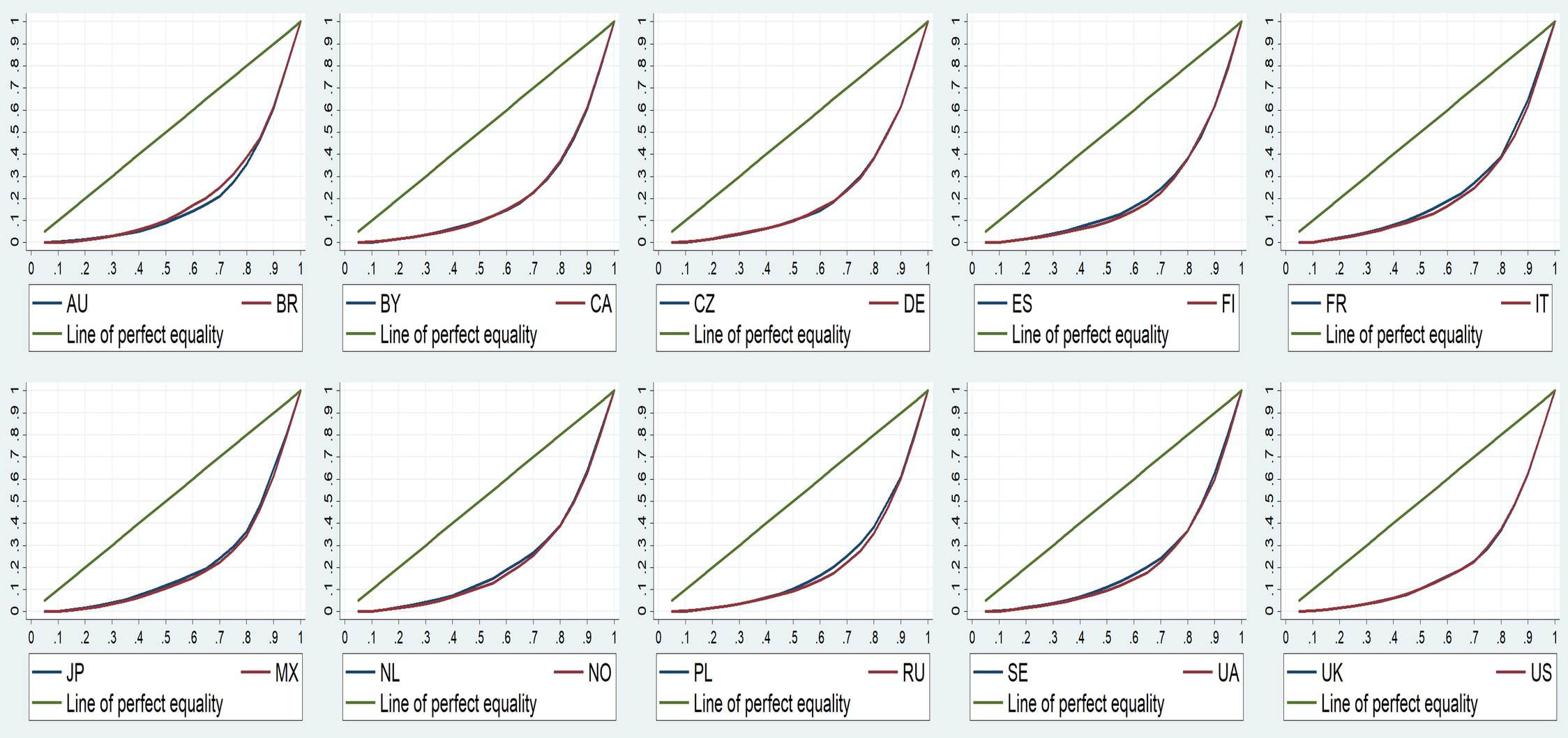

Supplement: S4 Fig — The y-axis indicates the percentage of the total listening frequencies and the y-axis indicates the percentage of genres. (TIF) [file pone.0208186.s004.tif]

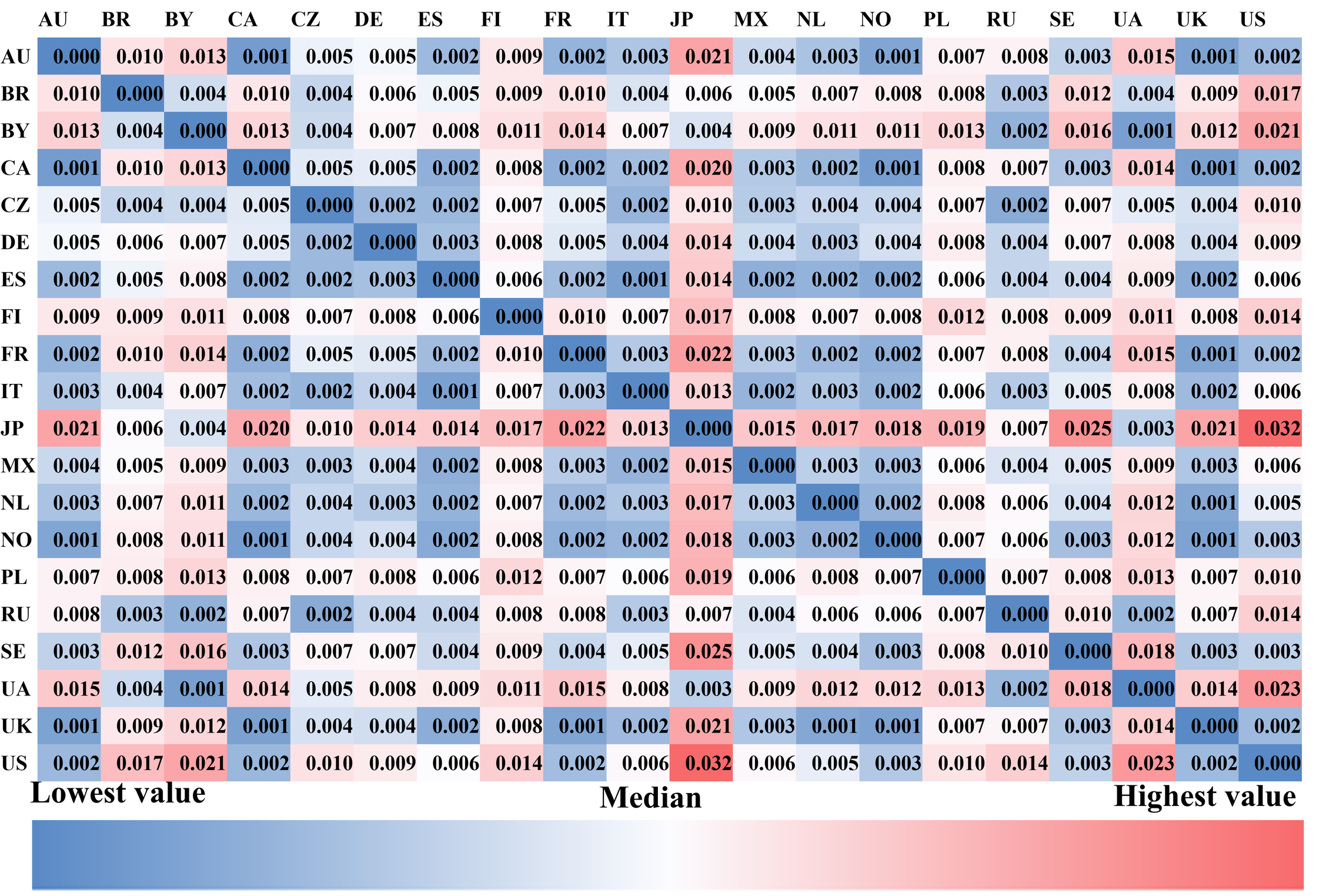

Supplement: S5 Fig — (TIF) [file pone.0208186.s005.tif]

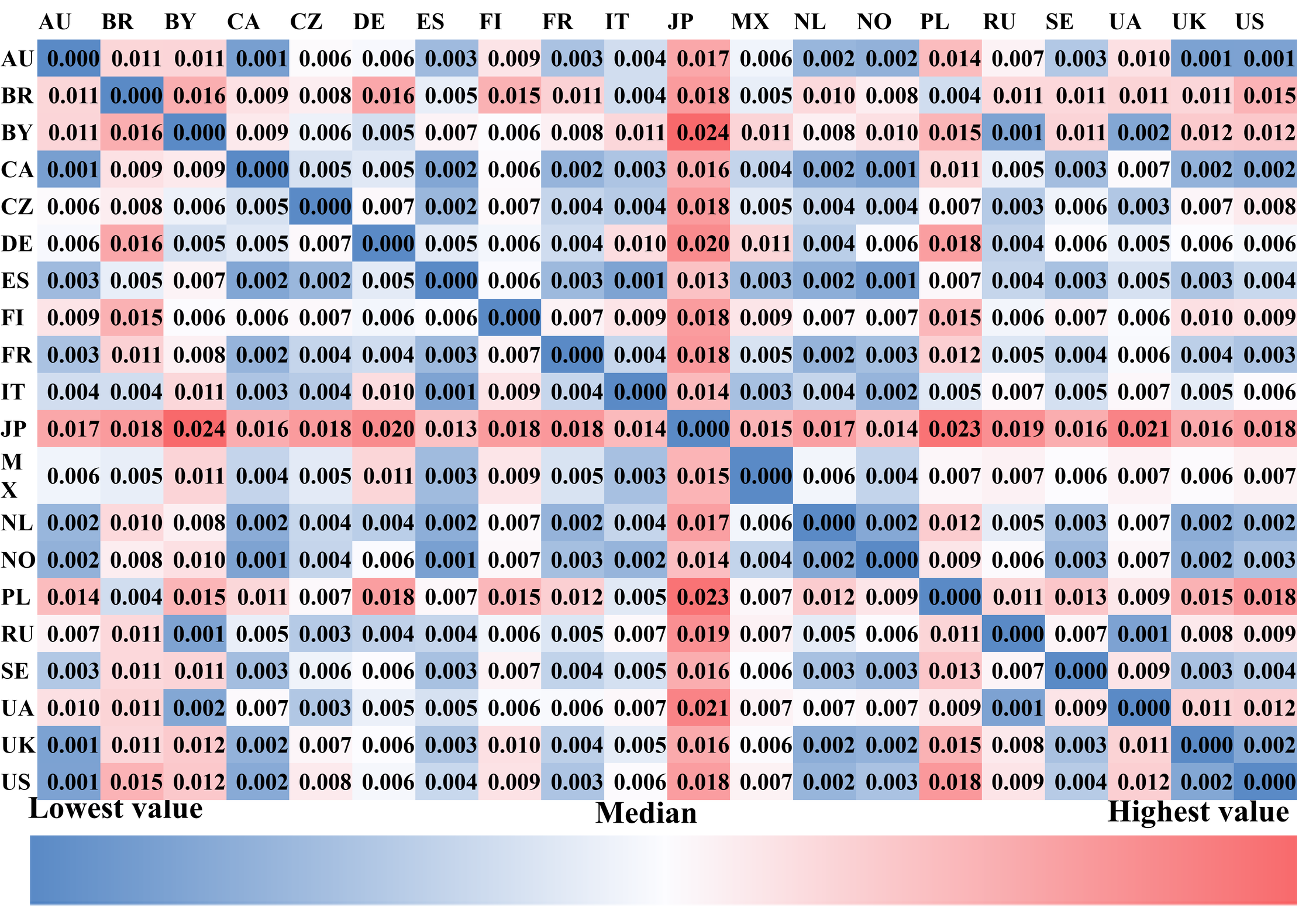

Supplement: S6 Fig — (TIF) [file pone.0208186.s006.tif]
