# Supplementary material for: The relation of culture, socio-economics, and friendship to music preferences: A large-scale, cross-country study
Source: PLoS One. 2018 Dec 14;13(12):e0208186. doi: 10.1371/journal.pone.0208186 (PMC6294554; doi:10.1371/journal.pone.0208186)
Supplement: S1 Table — (DOCX) [file pone.0208186.s007.docx]

**S1 Table. The distribution of users across the sampled countries and the populations of the countries.**

| Country | Country code | Number of users in  LFM-1b | Percent | Population | Index |
| --- | --- | --- | --- | --- | --- |
| The United States | US | 10,255 | 18.580 | 322583006 | 0.318 |
| Russia | RU | 5,024 | 9.120 | 142467651 | 0.353 |
| The United Kingdom | UK | 4,534 | 8.223 | 63489234 | 0.714 |
| Poland | PL | 4,408 | 7.991 | 38530725 | 1.144 |
| Brazil | BR | 3,885 | 7.043 | 202033670 | 0.192 |
| Finland | FI | 1,409 | 2.552 | 5443497 | 2.588 |
| Netherlands | NL | 1,375 | 2.493 | 16802463 | 0.818 |
| Spain | ES | 1,243 | 2.251 | 47066402 | 0.264 |
| Sweden | SE | 1,231 | 2.230 | 9631261 | 1.278 |
| Ukraine | UA | 1,143 | 2.070 | 45489600 | 0.251 |
| Canada | CA | 1,077 | 1.952 | 35158304 | 0.306 |
| France | FR | 1,055 | 1.911 | 66028467 | 0.160 |
| Australia | AU | 976 | 1.775 | 23630169 | 0.413 |
| Italy | IT | 974 | 1.761 | 59831093 | 0.163 |
| Japan | JP | 806 | 1.461 | 126999808 | 0.063 |
| Norway | NO | 750 | 1.360 | 5091924 | 1.473 |
| Mexico | MX | 705 | 1.280 | 123799215 | 0.057 |
| Czechia | CZ | 631 | 1.143 | 10521468 | 0.600 |
| Belarus | BY | 558 | 1.011 | 9307609 | 0.600 |

Notes: The percentage indicates the percentage of the users in each country to the total sample users in LFM-1b; Population refers to the entire population^[[1]](#footnote-1)^ of each country in 2014 (LFM-1b is up to 2014); Index is calculated by the following formula: (the number of users in a country in LFM-1b/the entire population of the country)$\times$10,000.

1. https://esa.un.org/unpd/wpp [↑](#footnote-ref-1)
