# Supplementary material for: The relation of culture, socio-economics, and friendship to music preferences: A large-scale, cross-country study
Source: PLoS One. 2018 Dec 14;13(12):e0208186. doi: 10.1371/journal.pone.0208186 (PMC6294554; doi:10.1371/journal.pone.0208186)
Supplement: S2 Table — (DOCX) [file pone.0208186.s008.docx]

**S2 Table. The values of the six cultural dimensions for the sampled countries.**

| Country | Country code | PDI | IDV | MAS | UAI | LTO | IND |
| --- | --- | --- | --- | --- | --- | --- | --- |
| Australia | AU | 38 | 90 | 61 | 51 | 21 | 71 |
| Brazil | BR | 69 | 38 | 49 | 76 | 44 | 59 |
| Belarus | BY | null | null | null | null | 81 | 15 |
| Canada | CA | 39 | 80 | 52 | 48 | 36 | 68 |
| Czech Republic | CZ | 57 | 58 | 57 | 74 | 70 | 29 |
| Germany | DE | 35 | 67 | 66 | 65 | 83 | 40 |
| Spain | ES | 57 | 51 | 42 | 86 | 48 | 44 |
| Finland | FI | 33 | 63 | 26 | 59 | 38 | 57 |
| France | FR | 68 | 71 | 43 | 86 | 63 | 48 |
| Italy | IT | 50 | 76 | 70 | 75 | 61 | 30 |
| Japan | JP | 54 | 46 | 95 | 92 | 88 | 42 |
| Mexico | MX | 81 | 30 | 69 | 82 | 24 | 97 |
| Netherlands | NL | 38 | 80 | 14 | 53 | 67 | 68 |
| Norway | NO | 31 | 69 | 8 | 50 | 35 | 55 |
| Poland | PL | 68 | 60 | 64 | 93 | 38 | 29 |
| Russia | RU | 93 | 39 | 36 | 95 | 81 | 20 |
| Sweden | SE | 31 | 71 | 5 | 29 | 53 | 78 |
| Ukraine | UA | null | null | null | null | 86 | 14 |
| the United Kingdom | UK | 32 | 80 | 67 | 35 | 38 | 67 |
| the United States | US | 40 | 91 | 62 | 46 | 26 | 68 |
