# Supplementary material for: The relation of culture, socio-economics, and friendship to music preferences: A large-scale, cross-country study
Source: PLoS One. 2018 Dec 14;13(12):e0208186. doi: 10.1371/journal.pone.0208186 (PMC6294554; doi:10.1371/journal.pone.0208186)
Supplement: S3 Table — (DOCX) [file pone.0208186.s009.docx]

**S3 Table.** **Overview of socio-cultural-economic aspects investigated in this study.**

| Category | Variables | Measures |
| --- | --- | --- |
| Geographic aspects | Geographic distance between countries | The length of the shortest curve between countries on a spheritic surface based on the latitudes and longitudes of the capital cities of the countries |
| Economic aspects | Economic distance between countries | The differences between countries in GDP per capita (PPP) |
| Cultural aspects | Cultural distance between countries | The differences between countries in the values of Hofstede’s six cultural dimensions for countries |
|  | Language distance between countries | The difference between the main language of countries based on the classification of languages in the language family tree |
| Social aspects | Friendship connection density between countries | The extent to which the users in any two countries are “friends” of each other on Last.fm |
