# Supplementary material for: The relation of culture, socio-economics, and friendship to music preferences: A large-scale, cross-country study
Source: PLoS One. 2018 Dec 14;13(12):e0208186. doi: 10.1371/journal.pone.0208186 (PMC6294554; doi:10.1371/journal.pone.0208186)
Supplement: S4 Table — (DOCX) [file pone.0208186.s010.docx]

**S4 Table. The QAP regression results for robustness check.**

| Variable | Model 1 | Model 2 |
| --- | --- | --- |
|  | (Album) | (Artist) |
| GEO | -0.027 | 0.184 |
| ECO | 0.264 | 0.167 |
| LAN | 0.429*** | 0.580*** |
| FRIEN | -0.002 *** | -0.069* |
| PDI | -0.314 | -0.012 |
| IDV | 0.195 | -0.001 |
| MAS | 0.074** | -0.030 |
| UAI | 0.191 | 0.040 |
| LTO | 0.321*** | 0.110** |
| IND | -0.028 | 0.029* |
| Adjusted R2 | 0.458 | 0.403 |
| N of Obs | 380 | 380 |

Notes: All coefficients presented are standardized coefficients; significance levels: ***: p < 0.001, **: p < 0.01, *: p < 0.05**.**
